# *In Vivo* Localization of *Iris yellow spot* Tospovirus (*Bunyaviridae*)-Encoded Proteins and Identification of Interacting Regions of Nucleocapsid and Movement Proteins

**DOI:** 10.1371/journal.pone.0118973

**Published:** 2015-03-17

**Authors:** Diwaker Tripathi, Gaurav Raikhy, Michael M. Goodin, Ralf G. Dietzgen, Hanu R. Pappu

**Affiliations:** 1 Department of Plant Pathology, P.O. Box 646430, Washington State University, Pullman, Washington, United States of America; 2 Department of Plant Pathology, University of Kentucky, Lexington, Kentucky, United States of America; 3 QAAFI, The University of Queensland, St. Lucia, Queensland, Australia; University of California, Riverside, UNITED STATES

## Abstract

**Background:**

Localization and interaction studies of viral proteins provide important information about their replication in their host plants. Tospoviruses (Family *Bunyaviridae*) are economically important viruses affecting numerous field and horticultural crops. *Iris yellow spot virus* (IYSV), one of the tospoviruses, has recently emerged as an important viral pathogen of *Allium spp*. in many parts of the world. We studied the in vivo localization and interaction patterns of the IYSV proteins in uninfected and infected *Nicotiana benthamiana* and identified the interacting partners.

**Principal Findings:**

Bimolecular fluorescence complementation (BiFC) analysis demonstrated homotypic and heterotypic interactions between IYSV nucleocapsid (N) and movement (NSm) proteins. These interactions were further confirmed by pull-down assays. Additionally, interacting regions of IYSV N and NSm were identified by the yeast-2-hybrid system and β-galactosidase assay. The N protein self-association was found to be mediated through the N- and C-terminal regions making head to tail interaction. Self-interaction of IYSV NSm was shown to occur through multiple interacting regions. In yeast-2-hybrid assay, the N- and C-terminal regions of IYSV N protein interacted with an N-terminal region of IYSV NSm protein.

**Conclusion/Significance:**

Our studies provide new insights into localization and interactions of IYSV N and NSm proteins. Molecular basis of these interactions was studied and is discussed in the context of tospovirus assembly, replication, and infection processes.

## Introduction

Thrips-transmitted tospoviruses cause serious losses in yield and quality of the produce of a wide range of field and horticultural crops in different parts of the world [1–3]. Total losses due to tospoviral diseases are estimated to be more than US$1 billion every year [4]. The genus *Tospovirus*, with the type species *Tomato spotted wilt virus* (TSWV) [5], consists of the plant-infecting members of the *Bunyaviridae*, a family that primarily comprises of animal-infecting viruses classified in the 4 genera *Orthobunyavirus*, *Hantavirus*, *Nairovirus*, and *Phlebovirus* [6].

Tospovirus particles are quasi-spherical in shape having diameter ranging from 80–110 nm that are enveloped by a lipid membrane. Virus particles contain tripartite negative-sense and ambisense single-stranded RNAs that are designated large (L), medium (M), and small (S) based on their sizes [7]. All RNA segments contain conserved and inverted complementary sequences at the 5` and 3` ends that enable the formation of a pan-handle structure. The tospovirus genome encodes the nucleocapsid protein (N), two enveloped glycoproteins (G_N_ and G_C_), silencing suppressor (NSs), movement protein (NSm) and an RNA dependent RNA polymerase (RdRp) [7–8]. The entire L RNA is in negative sense and codes for one large open reading frame (ORF) in the viral complementary (vc) strand. The RdRp (∼330kDa), encoded by the L RNA, is involved in replication and transcription of the viral genome [9].

The NSm and NSs proteins are encoded from the viral (v) strands of M and S RNAs, respectively [8–9]. The NSm protein has roles in the tubule structures formation that are required for cell-to-cell and systemic movement and development of symptoms [10–11]. The NSs protein is a suppressor of gene silencing to overcome the RNA interference defense mechanisms of plants [12–13] and is important for efficient accumulation of the virus in adult insects [14]. The structural glycoprotein (GP) precursor and nucleocapsid (N) are encoded from the vc strands of M and S RNAs, respectively [8–9]. Mature G_N_ and G_C_ glycoproteins are derived from proteolytic processing of GP. Both glycoproteins form protruding spikes on the surface of the lipid membrane envelope and are important for thrips transmission and vector specificity [9, 15].

*Iris yellow spot virus* (IYSV) is an economically important tospovirus impacting onion seed and bulb crops in the United States and several parts of the world [1–2, 16]. IYSV was first isolated as a new tospovirus from *Iris hollandica* in the Netherlands [17]. It was first described in the United States in 1989, infecting onion crops in Idaho [18]. In the US, the effect of disease is most damaging to onion seed crops since infection leads to lodging that could force the growers to abandon the crop resulting in 100% loss [1]. IYSV is of concern to the onion bulb and seed industries as few effective management options are currently available for control of the virus and/or the thrips vector [19].

To understand the molecular biology of IYSV in detail, we performed localization and interaction studies of its proteins. The intracellular localization of all IYSV proteins except RdRp was studied in both uninfected and IYSV-infected plants to observe if localization pattern of tospovirus proteins change in the presence of replicating virus. We also studied the interactions of the IYSV proteins in *Nicotiana benthamiana* cells using bimolecular fluorescence complementation (BiFC) technique to gain a better understanding of the association of IYSV proteins *in vivo*. The BiFC interaction results were further validated by maltose binding protein (MBP) pull down and yeast-2-hybrid assays. We also performed yeast-2-hybrid assays to identify interacting regions of IYSV N and NSm proteins.

## Materials and Methods

### Plant materials

Wild-type and transgenic *N*. *benthamiana* marker plants expressing autofluorescent markers (Red FP or Cyan FP) targeted to the nucleus (Histone H2B) were used [20]. Plants were grown in a greenhouse with 16 h day/8 h night cycle. A Washington isolate of IYSV from onion was maintained in *N*. *benthamiana* in a greenhouse under ambient conditions and was used as a source of inoculum for 4 to 6-weeks old plants. To perform the localization and BiFC interaction assays in infected leaves, symptomatic leaves of IYSV-inoculated plants were used for agroinfiltration at 10 to 14 days post inoculation (dpi).

### Amplification of IYSV genes from infected plants

Total RNA from IYSV-inoculated plants was isolated using RNeasy plant mini kit (Qiagen), and cDNA was generated using Superscript II reverse transcriptase (Invitrogen) and gene-specific primers. IYSV ORFs for the N, NSm, NSs, G_N_ and G_C_ proteins were amplified by PCR using Platinum *Pfx* DNA polymerase (Invitrogen) and *attB* sequence-flanked virus-specific primers (Table 1). IYSV-specific primer sequences were designed from the published sequences of IYSV isolates (GenBank accession numbers JQ973067 /AF001387.1 /AF214014.1). IYSV blunt-end amplicons were cloned into pDONR221 or pENTR-D-TOPO vectors (Invitrogen) and three clones were sequenced in both directions using M13 forward and reverse primers and gene-specific primers.

**Table 1:** List of primers used for bimolecular fluorescence complementation and pull down assays.

^a^Primer name	^b^ Primer sequence (5’-3’)	Size (bp)	Tm (°C)
IYSV N_F	caccATGTCTACCGTTAGGGTGAAA	819	51
IYSV N_R	ATTATATCTATCCTTCTTGGAG		
IYSV NSm_F	ggggacaagtttgtacaaaaaagcaggcttcATGTCTCTCCTAACTAACGTG	936	60
IYSV NSm_R	gggggaccactttgtacaagaaagctgggtcTACTTCATTAAATCTGTTCTCGTT		
IYSV NSs_F	ggggacaagtttgtacaaaaaagcaggcttcATGTCTACCGTTAGGACTAC	1328	55
IYSV NSs_R	gggggaccactttgtacaagaaagctgggtcCTGCAGCTCTTCTACAGTAA		
IYSV G_N__F	caccATGAATTTACAATATCTACTACTC	1347	54
IYSV G_N__R	TGCCAAACTAGATGGTAT		
IYSV G_C__F	caccATGCCCAGGCAATCT	1575	54
IYSV G_C__R	AAAATCTAAAGGGAACTGA		

^a^ Primer names with the suffix ‘F’ are forward primers, while those with ‘R’ are reverse primers.

^b^ Sequence of primer, where the underlined nucleotides are *att* sequence for the Gateway cloning.

### Transient expression of fusion proteins and BiFC assay

For protein localization and BiFC studies, IYSV proteins were expressed in wild-type, and marker *N*. *benthamiana* plants. In brief, full-length ORF entry clones of IYSV without a stop codon were recombined into binary destination pSITE or pSITE II vectors. pSITE-2CA [green fluorescent protein (GFP) fusions] vectors were used for localization studies [20–23].

For BiFC interaction assays, IYSV proteins were fused to the amino- and carboxy-terminal portions of yellow fluorescent protein [pSITE-BiFC-nEYFP-C1 and pSITE-BiFC-cEYFP-C1] and pSITE-BiFC-N1 vectors. Expression of fusion constructs was tested by Western blotting using GFP and YFP antibodies (Santa Cruz Biotechnology Inc.). Interactions were tested in all pairwise combinations and orientations as described previously [24]. As a non-binding control, Glutathione-S-transferase (GST) was used in all studies. For co-localization studies, organelle markers that were developed as binary plasmids [25] were obtained from the Arabidopsis Biological Research Center (ABRC; Ohio). The markers used in this study were ER-ck or -rk (endoplasmic reticulum with cyan or red fluorescent protein; ER-CFP or ER-RFP). Recombinant pSITE vectors with IYSV genes and organelle markers were transformed into *Agrobacterium tumefaciens* LBA4404. Transformed agroclones were infiltrated into *N*. *benthamiana* marker plants as described [26]. Agroinfiltrated plants were kept under constant illumination at 25°C. After 48-h incubation, water-mounted sections of leaf tissue were examined by Leica confocal laser scanning microscope (Leica Microsystems). A minimum three leaves for each expression construct were examined and high-quality images were obtained as described in [26].

### Pull down assay

Maltose binding protein (MBP) pull down assay was performed as described [27] with some modifications. Briefly, IYSV N and NSm amplicons were cloned into pDONR221 or pENTR-D-TOPO vectors (Invitrogen). The entry clones were recombined into GW pMAL-c2X and pDEST15 vectors (Invitrogen). Pull-down assays were performed by mixing “bait” (MBP alone or fused to IYSV N or NSm) crude protein extract with “prey” (protein extract, from *E*. *coli* expressing GST with IYSV proteins or empty expression vector) crude protein extract in a microcentrifuge tube. For each treatment, “Load”, “Flow-Through” and “Elution” samples were made and were separated on a 10% SDS-polyacrylamide gel and transferred to a PVDF membrane (Millipore) following manufacturer’s instructions. The blot was probed with anti-GST monoclonal antibodies (1:2000) (Sigma) and with HRP conjugated secondary antibody (1:1000) (Sigma). Images were acquired within 5 minutes of ECL (GE healthcare) addition using VersaDoc imaging system (Bio-Rad) according to the manufacturer’s specifications.

### Yeast-2-hybrid (Y2H) and β-galactosidase assays

Yeast-2-hybrid assays were performed as described [28]. Plasmids pEG202 and pJG4-5, as well as yeast strain EGY48 harboring pSH18-34, were a gift from Scott Leisner (University of Toledo, OH). IYSV N and NSm genes were divided into four and three regions respectively. Reverse primers were designed with termination codons. All regions were amplified with *Pfu* DNA polymerase by the Gateway primer sequences (Table 2). Amplicons were cloned into *att*P-containing pDONR221 or pENTR-D-TOPO vectors using the Gateway cloning system (Invitrogen) and the entry clones were sequenced as described earlier. The confirmed entry clones were recombined into binary yeast plasmids with either the LexA DNA-binding domain-encoding region of pEG202 or the DNA sequence coding for the B42 activation domain in pJG4-5 [29–30].

**Table 2:** List of primers used for yeast-2-hybrid assays.

^a^Primer name	^b^ ^,^ ^c^ Primer sequence (5’-3’)	Length (aa)	Tm (°C)
IYSV N_F	caccATGTCTACCGTTAGGGTGAAAC	1–273	51
IYSV N_R	TTAATTATATCTATCCTTCT		
IYSV N2_F	caccATGTCTACCGTTAGGGTGAAAC	1–90	51
IYSV N2_R	TCTAACCTCCTGAATG*T*CA		
IYSV N3_F	caccTGGACATTCAGGAGGTTAGA	91–180	51
IYSV N3_R	CTGCTTATACCGAGTGCTT*A*		
IYSV N4_F	caccGAAGCACTCGGTATAAGCAG	181–220	51
IYSV N4_R	GTGCATTCAGTGAGGATCT*A*		
IYSV N5_F	caccAAGATCCTCACTGAATGCAC	221–273	51
IYSV N5_R	TTAATTATATCTATCCTTCT		
IYSV NSm_F	ggggacaagtttgtacaaaaaagcaggcttcATGTCTCTCCTAACTAACGT	1–312	60
IYSV NSm_R	gggggaccactttgtacaagaaagctgggtcTCATACTTCATTAAATCTGT		
IYSV NSm2_F	ggggacaagtttgtacaaaaaagcaggcttcATGTCTCTCCTAACTAACGT	1–160	60
IYSV NSm2_R	gggggaccactttgtacaagaaagctgggtcTTTCTT*A*AGGTGTCATCTTA		
IYSV NSm 3_F	ggggacaagtttgtacaaaaaagcaggcttcAAAGATGATTCCCTTATTGG	100–200	60
IYSV NSm3_R	gggggaccactttgtacaagaaagctgggtcTAGTCAA*CTA*AAGCTGCATA		
IYSV NSm4_F	ggggacaagtttgtacaaaaaagcaggcttcAATTTGACTAGTAACGAAAA	201–312	60
IYSV NSm4_R	gggggaccactttgtacaagaaagctgggtcTCATACTTCATTAAATCTGT		

^a^ Primer names with the suffix ‘F’ are forward primers, while those with ‘R’ are reverse primers.

^b^ Sequence of primer, where the underlined nucleotides are *att* sequence for the Gateway cloning

^c^ The italicized underlined nucleotides are stop codan sites.

The recombinant plasmids were amplified in *E*. *coli* and subsequently introduced into *Saccharomyces cerevisiae* EGY48 harboring the pSH18-34 β-galactosidase reporter plasmid using a lithium acetate yeast transformation procedure [28–31]. First, the pEG202 plasmids harboring either full-length N and NSm genes of IYSV or their various fragments were combined with yeast strain EGY48 containing pSH18-34 and were selected on synthetic defined (SD)/-Ura-His medium (Clontech laboratories). Individual colonies were used to establish yeast lines harboring recombinant pEG202 plasmids. Recombinant pJG4-5 plasmids were then introduced into these lines. The transformants were grown at 30°C on agar plates containing either SD Base/Gal/Raf/-Ura-His-Trp (+L) or SD Base/Gal/Raf/-Ura-His-Trp-Leu (-L) (Clontech lab.) for 3–5 days. Vector pJG4-5 without a gene insert was used as a negative control for each set of yeast transformations. β-galactosidase assays were performed to measure reporter gene activity as described in [28–29].

## Results

### Localization of IYSV proteins in *N*. *benthamiana* leaves

**Fig 1:**
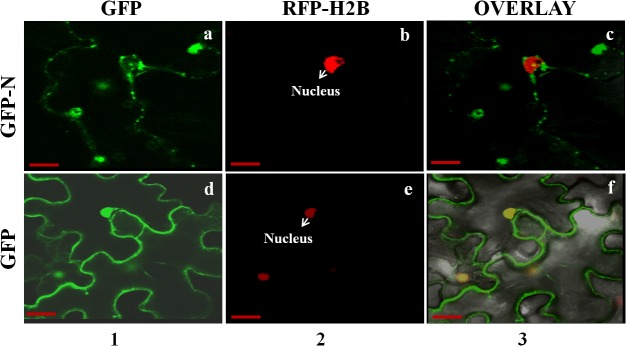
Localization of *Iris yellow spot virus* (IYSV) nucleocapsid (N) protein in epidermal cells of uninfected transgenic *Nicotiana benthamiana* plants containing the red nuclear marker histone 2B (RFP-H2B). Confocal micrographs represent IYSV fusion proteins to the C-terminus of green fluorescent protein (GFP). Columns from left to right show GFP-gene fusion or free GFP (1), RFP-H2B (2), and the overlay of the images (3). (a-c). IYSV N fusion with GFP in RFP-H2B plants; (d-f). Free GFP in RFP-H2B plants. Each micrograph represents minimum 50 cells that were examined for localization. Scale bar = 20μm.

To study the localization pattern of IYSV proteins, each gene was fused with GFP at both N- and C-termini and agroinfiltrated in wild-type and marker RFP-H2B plants. Each fusion protein was also agroinfiltrated in the IYSV-inoculated leaves of *N*. *benthamiana* plants. Confocal microscopy was performed on the leaf sections of agroinfiltrated plants. As a control, GFP without any fusion was agroinfiltrated in plants. GFP alone localized to the nucleus and cell periphery (Fig. 1). Our localization studies suggested that the IYSV N protein localizes in the cytoplasm and the cell periphery of wild-type as well as marker RFP-H2B plants as aggregates (Fig. 1).

**Fig 2:**
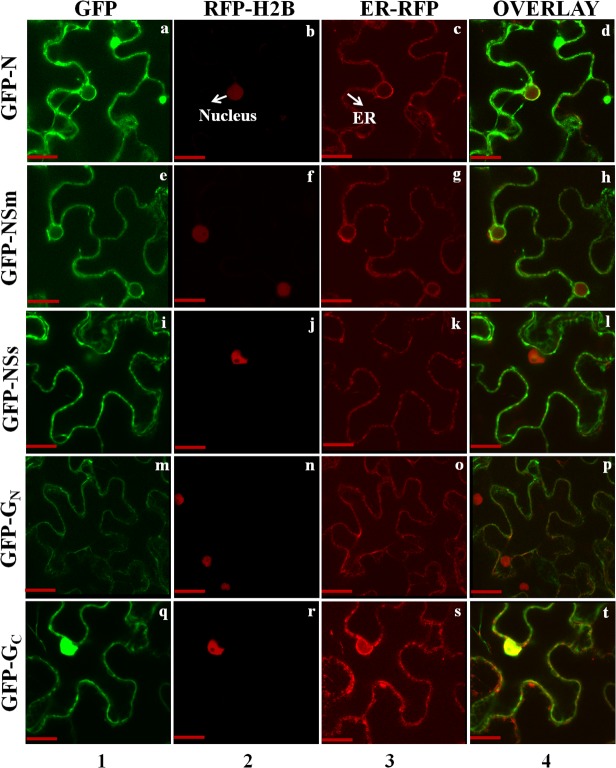
Localization of *Iris yellow spot virus* (IYSV) proteins, in epidermal cells of IYSV-infected transgenic *Nicotiana benthamiana* plants containing the red nuclear marker histone 2B (RFP-H2B), and red endoplasmic reticulum marker (ER-RFP). Confocal micrographs represent IYSV fusion proteins to the C-terminus of green fluorescent protein (GFP). Columns from left to right show GFP-gene fusion (1), RFP-H2B (2), ER-RFP (3), and the overlay of the images (4). (a-d) GFP-IYSV N co-expression with RFP-H2B and ER-RFP; (e-h) GFP-IYSV NSm co-expression with RFP-H2B and ER-RFP; (i-l) GFP-IYSV NSs co-expression with RFP-H2B and ER-RFP; (m-p) GFP-IYSV G_N_ co-expression with RFP-H2B and ER-RFP; (q-t) GFP-IYSV G_C_ co-expression with RFP-H2B and ER-RFP. Each micrograph represents a minimum of 50 cells that were examined for localization. Scale bar = 20μm.

In RFP-H2B plants infected with IYSV, IYSV N protein localized in the cell periphery and co-localized with the endoplasmic reticulum (ER) when expressed with a binary ER-RFP marker (Fig. 2 A-D and S1 Fig.). IYSV NSm and NSs were fused with GFP and expressed in wild-type and RFP-H2B transgenic plants. Both these non-structural proteins localized in the cell periphery and colocalized with the ER-RFP marker. Similar expression profiles of IYSV NSm and NSs were observed in infected plants (Fig. 2 E-L and S1 Fig.). To study the localization of IYSV glycoproteins, the N- and C-termini of glycoprotein precursors (G_N_ and G_C_) were expressed separately in RFP-H2B plants. GFP fusions of the mature glycoproteins, G_N_ and G_C_, in uninfected and infected RFP-H2B plants also localized to the cell periphery and co-localized with ER. Although, GFP-G_N_ localized exclusively to the cell periphery, GFP-G_C_ localized to both the cell periphery and the nucleus. IYSV G_N_ and G_C_ were also found to be colocalized with ER-RFP marker (Fig. 2 M-T and S1 Fig.). IYSV protein fusion with RFP formed similar localization patterns in the cell periphery and colocalized with the ER network (data not shown).

### Homotypic and heterotypic interactions of IYSV proteins

**Fig 3:**
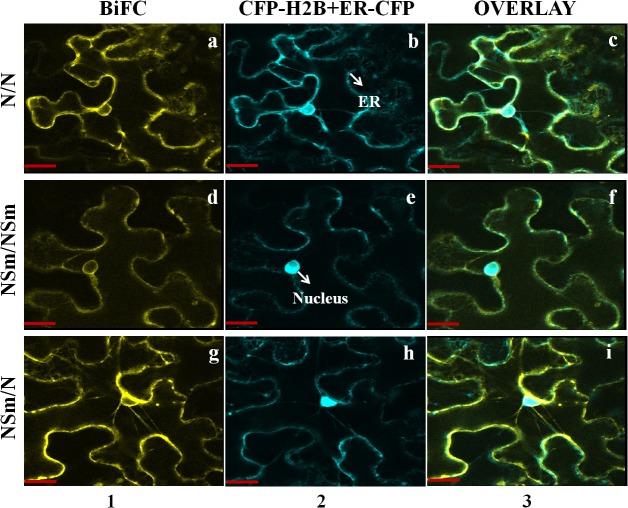
*In planta* interactions of *Iris yellow spot virus* proteins as examined by bimolecular fluorescence complementation (BiFC) assay. Interaction assays were performed in leaf epidermal cells of IYSV-infected transgenic *Nicotiana benthamiana* plants expressing cyan fluorescent protein fused to the nuclear marker histone 2B (CFP-H2B), and cyan endoplasmic reticulum (ER-CFP) marker. Column 1 shows BiFC, column 2 shows localization of CFP-H2B and ER-CFP (nucleus and ER), and column 3 shows a merge of all panels (overlay). The first and second proteins mentioned in each pair of interactors were expressed as C-terminal fusions to the amino-terminal half of YFP and as C-terminal fusions to the carboxy-terminal half of YFP respectively. A set of positive interactions is shown here after testing interactions in all pairwise combinations: (a-c) N/N, (d-f) NSm/ NSm, (g-i) NSm/N. Each micrograph represents a minimum of 50 cells that were examined for interaction. Scale bar = 20μm.

All IYSV structural and non-structural proteins, except RdRP were tested for interaction using the BiFC approach in all pairwise combinations. IYSV proteins were expressed in binary pSITE-BiFC vectors and co-agroinfiltrated into wild-type *N*. *benthamiana* and marker CFP-H2B plants. IYSV N protein interactions with itself and other structural and non-structural proteins were tested. Homotypic or self-interaction of the N and NSm proteins and heterotypic interaction between the N and NSm proteins were detected by BiFC. Interactions between IYSV N and NSm were observed in both orientations. All positive interactions were found to occur in the cell periphery when proteins were co-expressed with ER-CFP marker in infected CFP-H2B plants (Fig. 3 A-I). The self-interaction of N protein and its interaction with NSm led to the formation of some aggregates in the cell periphery suggesting potential multi-molecule complexes. All possible combinations of N and NSm proteins were tested for interactions. However, only combinations that had proteins fused to C-terminal region of YFP showed positive interactions (S2 Fig.). Interaction of N with NSs and glycoproteins were not observed in our BiFC assays when they were expressed from the BiFC-C1 and BiFC-N1vectors. None of the IYSV proteins tested interacted with GST in either orientation (data not shown).

**Fig 4:**
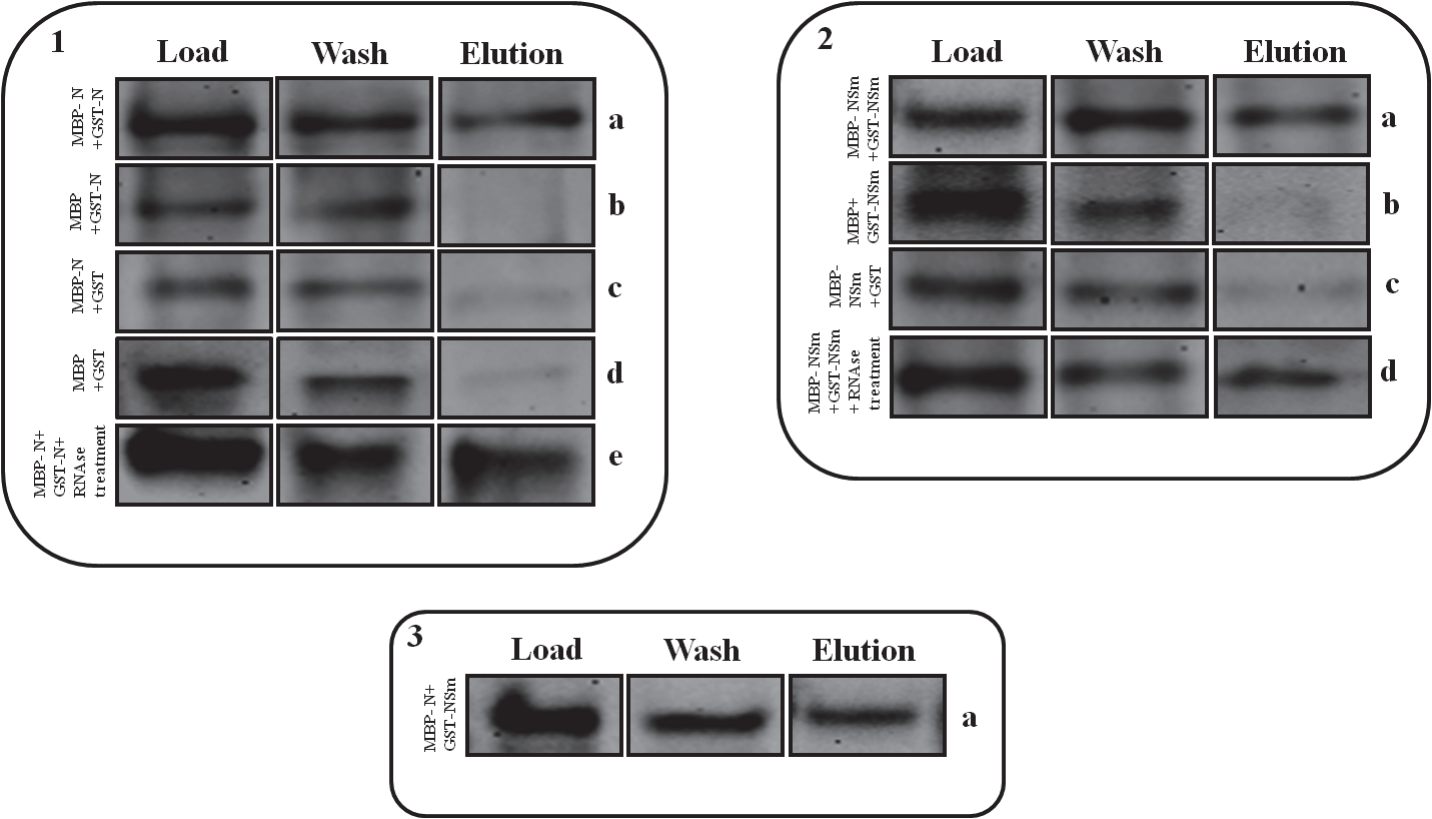
Maltose binding protein pull-down assays of *Iris yellow spot virus* (IYSV) proteins. Self-interaction of nucleocapsid; N (1) and movement; NSm (2) proteins, and cross-interaction of IYSV N and NSm proteins (3). ‘Load’ above the column represents the protein content initially loaded to the MBP column; ‘wash’ above the column comprises of the protein not adhering to the column; ‘elution’ was the material that attached to the column and was desorbed by the addition of maltose. Proteins were subjected to protein gel blot analysis and probed with anti-GST primary antibody. (1.a) MBP-tagged N mixed with GST-tagged N. (1.b) MBP alone mixed with GST-tagged N. (1.c) MBP-tagged N mixed with GST alone. (1.d) GST alone mixed with MBP alone. (1.e) The same combination as 1.a, except that proteins were first subjected to RNase treatment. (2.a) MBP-tagged NSm mixed with GST-tagged NSm. (2.b) MBP alone mixed with GST-tagged NSm. (2.c) MBP-tagged NSm mixed with GST alone. (2.d) The same combination as 2.a, except that proteins were first subjected to RNase treatment. (3.a) MBP-tagged N mixed with GST-tagged NSm.

Homotypic and heterotypic interactions of IYSV N and NSm proteins were biochemically confirmed by MBP pull down assays. Approximately half of the GST-N or -NSm bound to the MBP-N or -NSm resin and bound partners were eluted as a complex (Fig. 4, panel a). However, no cross-reacting bands were observed in the elution fraction of samples containing GST-N or -NSm added to the amylose resin expressing MBP alone, empty pDEST15 expressing GST alone, MBP-N or -NSm, or when crude samples of both empty vectors were used for pull down analyses (Fig. 4). Since TSWV N contains two RNA-binding domains and its NSm interacts with RNA [4, 32], it is possible that viral RNA bridging the interactions could also mediate IYSV N and NSm self-interaction. To test this possibility, proteins were treated with RNase A. RNase A treatment had no effect on IYSV N protein self-interaction indicating that this interaction was independent of RNA (Fig. 4). Taken together, BiFC and pull-down data indicate that the IYSV N and NSm self-associate and IYSV N interacts with its NSm protein.

### Interacting regions of IYSV N and NSm proteins

**Fig 5:**
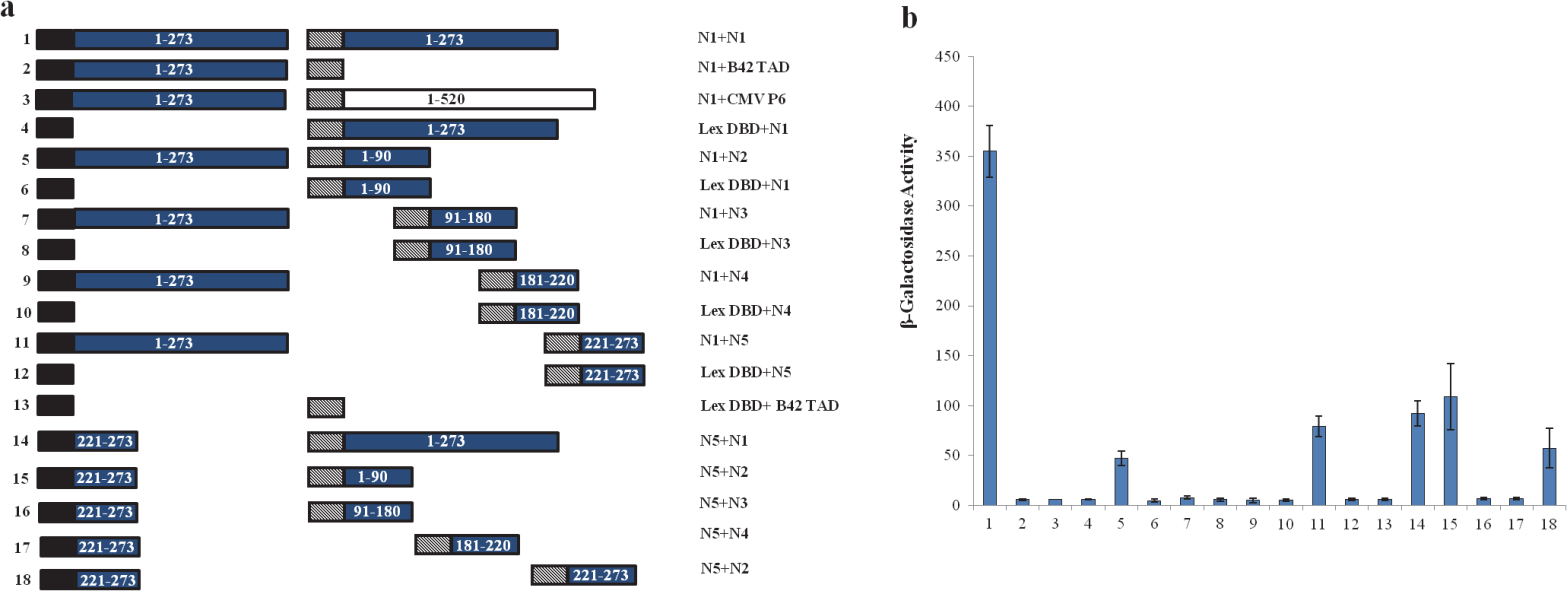
Self-interaction of *Iris yellow spot virus* (IYSV) nucleocapsid (N) protein. (a) Schematic diagram of the constructs tested for leucine independent growth and β-galactosidase activity. Black box, LexA DBD in pEG202; hatched box, B42 TAD in pJG4-5; blue boxes, IYSV N (N1-full length, amino acids 1–273; N2, amino acids 1–90; N3, amino acids 91–180; N4, amino acids 181–220; N5, amino acids 221–273), white box, full length *Cauliflower mosaic virus* P6 (amino acids 1–520). Numbers to the left of each pair of constructions correspond to the β-galactosidase assays shown in (b). (b) β-galactosidase activity of yeast transformants expressing constructs as shown in (a).

To identify the regions within the N and NSm that are responsible for the interactions, N and NSm were divided into four and three regions, respectively whose coding regions were generated by PCR. Amplicons were inserted into yeast two-hybrid vectors and tested for interactions. The only yeast transformants showing leucine-independent growth and β-galactosidase activity were those expressing N protein fused to the DBD and portions of N protein containing the N-terminal (1–90 aa; N2) and C-terminal (221–273 aa; N5) regions connected to the TAD (Fig. 5). None of the remaining co-transformants was able to grow on leucine-deficient media and all lacked β-galactosidase activity. To identify the portion (s) of N that the N2 and N5 regions interacted with, IN5 was tested against the other N protein regions. IN5 region bound efficiently only to N2 and itself (Fig. 5). Hence, only the yeast transformants expressing the N2 and N5 terminal regions of IYSV N fused to both the LexA DBD and B42 TAD showed leucine-independent growth and β-galactosidase activity.

**Fig 6:**
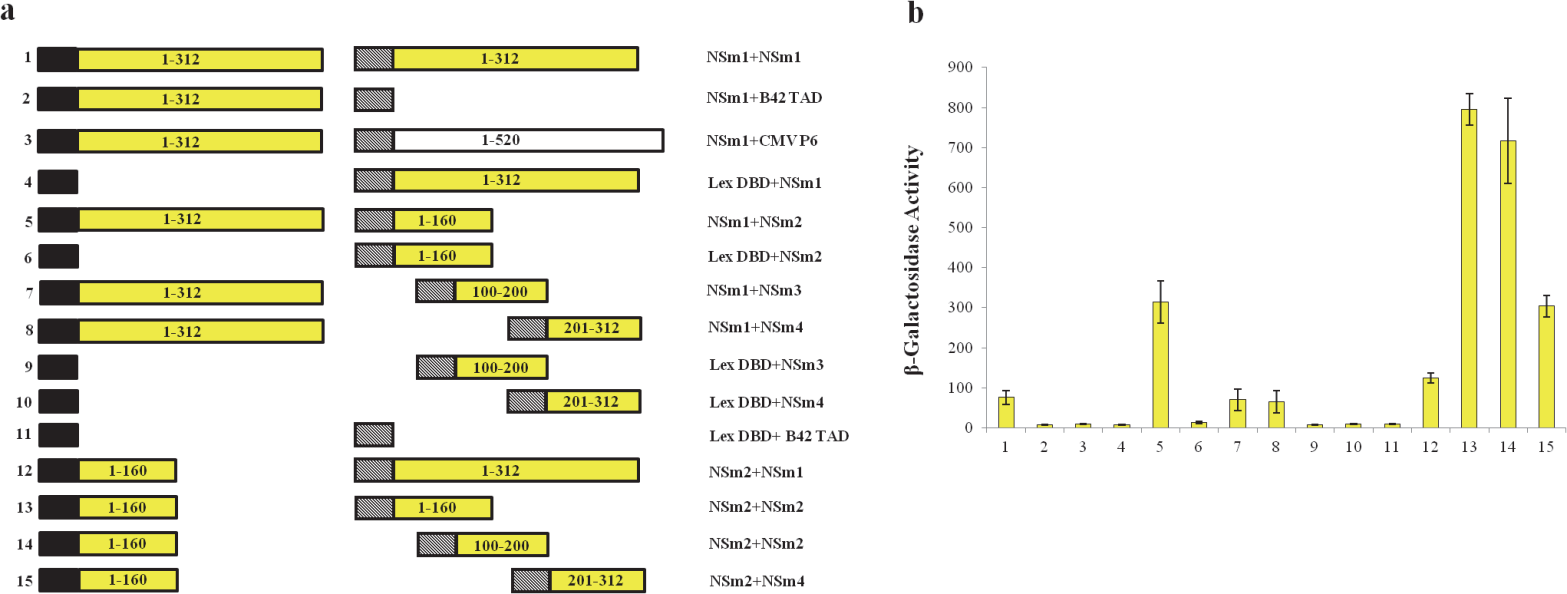
Self-interaction of *Iris yellow spot virus* (IYSV) movement protein. (a) Schematic diagram of the constructs tested for leucine independent growth and β-galactosidase activity. Black box, LexA DBD in pEG202; hatched box, B42 TAD in pJG4-5; yellow boxes, IYSV NSm (NSm1-full length, amino acids 1–312; NSm2, amino acids 1–160; NSm3, amino acids 100–200; NSm4, amino acids 201–312); white box, full length *Cauliflower mosaic virus* P6 (amino acids 1–520). Numbers to the left of each pair of constructions correspond to the β-galactosidase assays shown in (b). (b) β-galactosidase activity of yeast transformants expressing constructs as shown in (a).

In case of NSm, the interacting regions were those expressing NSm fused to the DBD and expressing portions of NSm containing the N-terminal (1–160 aa; NSm2), middle (100–200 aa; NSm3 and C-terminal (201–312 aa; NSm4) regions connected to the TAD (Fig. 6). INSm2 region bound efficiently to all other regions of IYSV NSm (Fig. 6).

**Fig 7:**
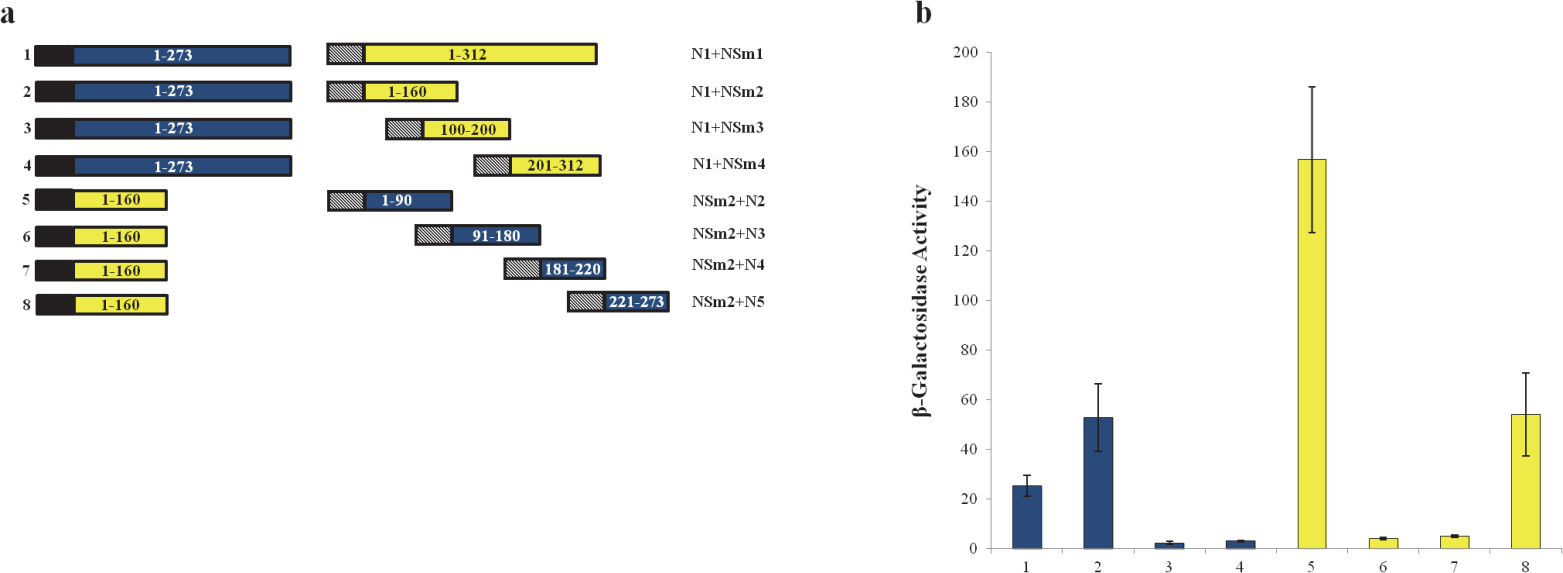
Heterotypic interaction of *Iris yellow spot virus* (IYSV) nucleocapsid (N) and movement (NSm) proteins. (a) Schematic diagram of the constructs tested for leucine independent growth and β-galactosidase activity. Black box, LexA DBD in pEG202; hatched box, B42 TAD in pJG4-5; blue boxes, IYSV N (N1-full length, amino acids 1–273; N2, amino acids 1–90; N3, amino acids 91–180; N4, amino acids 181–220; N5, amino acids 221–273); yellow boxes, IYSV NSm (NSm1-full length, amino acids 1–312; NSm2, amino acids 1–160; NSm3, amino acids 100–200; NSm4, amino acids 201–312). Numbers to the left of each pair of constructions correspond to the β-galactosidase assays shown in (b). (b) β-galactosidase activity of yeast transformants expressing constructs as shown in (a).

In cross-interaction assays, IYSV N fused to the DBD interacted with a portion of NSm containing the N-terminal (1–160 aa; NSm2) region connected to the TAD (Fig. 7). When NSm2 was tested against different IYSV N protein regions, only N-terminal region of IYSV N (1–90 aa; N2) and a C-terminal region of IYSV N (221–273 aa; N5) bound tightly with NSm2 and showed leucine-independent growth and β-galactosidase activity (Fig. 7).

In summary, we have shown that IYSV N and NSm proteins specifically self-associate and associate with each other. The N protein self-association is likely mediated through N-terminal and C-terminal regions in a head to tail configuration. The NSm self-interaction occurs through interactions involving all regions, especially the N-terminal region. IYSV N interacts with NSm through N-and C-terminal regions of IYSV N and N-terminal region of IYSV NSm.

## Discussion

Based on the N and NSm protein sequences, tospoviruses have been grouped into “New World” or American (e.g. TSWV, INSV) and “Old world” or Eurasia (e.g. IYSV) species [4, 33–34]. IYSV is currently present in most of the *Allium*-growing regions of the world and is a constraint to onion production [1]. The intracellular localizations and interactions of two tospoviruses, TSWV and INSV, were the subject of previous studies [4, 9–11, 23, 32]. As IYSV is a distinct and economically important species of the Eurasia group in the *Tospovirus* genus, we studied the localization and interaction patterns of the IYSV proteins in epidermal leaf cells of wild-type and marker RFP/CFP-H2B plants. Additionally, we investigated the localization and interactions of IYSV proteins in infected cells to determine if there is any change in localization in the presence of replicating virus. All studied IYSV proteins were found to localize in the cell periphery and endoplasmic reticulum (ER).

To study the *in planta* localization of IYSV N protein, it was fused with GFP and the fusion protein was found to be localized in the cytoplasm and cell periphery as aggregates when expressed following agroinfiltration of wild-type and RFP-H2B plants. Previous studies on TSWV and INSV proteins have shown a similar localization pattern of the N protein in *N*. *benthamiana* leaves [4, 23, 32]. When IYSV N protein fused with GFP was agroinfiltrated into IYSV-infected wild-type and RFP-H2B plants, it localized in the cell periphery. This type of localization pattern was not observed in uninfected TSWV and INSV N proteins and was only seen in our study in IYSV-infected plants. The co-infiltration of GFP fusion of IYSV N and binary vector containing ER-RFP marker in RFP-H2B plants formed similar localization pattern in the cell periphery and the N protein co-localized with ER. Recently, it was shown that the INSV N protein had a similar co-localization pattern when expressed as RFP fusion with the ER targeted GFP in 16c transgenic *N*. *benthamiana* plants [23]. This co-localization pattern was only observed in live plant cell imaging of INSV and IYSV. TSWV N protein in BHK21 cells or in tobacco protoplasts did not show similar co-localization pattern [9, 35]. Recently the co-localization of TSWV N was shown with actin filaments and ER using live plant cell imaging of intact leaves, which also confirms the co-localization of tospovirus N protein with the ER network [36].

In previous studies, the NSm protein of TSWV and INSV was shown to be localized on the cell periphery along the ER network suggesting some additional functions of NSm [5, 32]. Similar localization pattern of IYSV NSm was observed in our studies in uninfected and infected RFP-H2B plants. In addition to N and NSm, all other IYSV proteins (NSs, G_N_ and G_C_) were also found to be localized to the cell periphery with the ER network, which confirms the cytoplasmic location for replication of tospoviruses in infected cells [5].

Homotypic interaction of tospovirus N proteins has been previously reported in TSWV using a variety of interaction assays [4, 5, 37]. *In planta* homotypic interaction has also been reported for Capsicum chlorosis tospovirus and INSV using BiFC [23, 38]. Our studies on IYSV N protein interaction by BiFC and pull down assays showed a similar pattern of homotypic interaction of the N protein. Homotypic interaction and multimerization of the N protein have been speculated as a prerequisite in tospovirus replication [4]. IYSV N protein localization and interaction with itself in the cell periphery suggests a shift in its localization from the cytoplasm to cell periphery where it interacts with itself and other proteins in infected plants.

The NSm protein of tospoviruses has been reported to have typical characteristics of virus movement proteins as it supports cell-to-cell movement by formation of tubule structures [10]. Previous studies on TSWV NSm have reported its transient expression early in systemic infection, its interaction with TSWV RNA and N protein in the cytoplasm, and its localization close to plasmodesmata, cell wall, and cytoplasmic membranes [11]. Our study shows the localization and homotypic interaction of IYSV NSm in the cell periphery at ER sites in infected cells. Homotypic interaction of the NSm proteins was recently reported in INSV by using BiFC, but it was not found in TSWV suggesting that some interactions can only be observed by live cell imaging [23].

Homotypic interaction of the NSm proteins on the cell periphery at ER sites confirms their role in intercellular trafficking as a multimeric complex. Heterotypic interaction between the N and NSm has been reported in TSWV and INSV and has been proposed as a prerequisite in tubule-guided movement of tospoviruses [23, 32]. The interaction between IYSV N and NSm was also shown in this study using BiFC, pull down, and yeast-2-hybrid assays. Interaction between TSWV N and NSm that was reported previously [32], was validated by using BiFC and we found similar patterns of interaction as shown in IYSV (S3 Fig.). All these findings confirm that nucleocapsid and movement proteins of tospoviruses interact with each other in infected plants in the cell periphery at the ER.

In our study, the IYSV NSm protein interacted with the N protein and with itself, which might assist in its function in ribonucleoprotein (RNP) binding and tubule formation. Tospovirus-encoded NSm has been reported to express early in the systemic infection and aggregate with the nucleocapsid protein in the cytoplasm of host cells [10, 11]. Thus, interaction between NSm and N proteins and with tospoviral RNA explains a molecular basis for specific recognition of nucleocapsid structures. N/NSm interaction also suggests cellular trafficking of these proteins with different host proteins [5, 23, 32, 39]. The molecular basis of TSWV N/N interaction has been well studied. Homotypic interaction of the TSWV N protein is reportedly mediated by its N-terminal and C-terminal regions and results from a head-to-tail configuration [4]. However, molecular basis of tospovirus NSm/NSm and N/NSm interactions has not been studied. We used the yeast-2-hybrid system to identify the regions of IYSV N and NSm responsible for these interactions. Our data suggest that the homotypic interaction of IYSV N is mediated by an N-terminal (1–90 aa) and a C-terminal (221–273 aa) region, as shown in self-interaction of TSWV N [4]. Moreover, these N protein regions also interact with an N-terminal region of NSm. Hence, these head-to-tail interactions of N- and C-terminal domains play roles in self-interaction and multimerization of the N protein of tospoviruses. This interaction may also be of crucial significance for N/NSm interaction, which is a prerequisite for tubule-guided movement of tospovirus N protein [10]. IYSV NSm-NSm interaction in our study involves all three regions (N-, middle, and C-) of the NSm protein. These regions in TSWV NSm have been reported to be involved in tubule formation, movement and symptomatology of the “new world” tospoviruses [11].

The NSs protein of tospoviruses has been reported to obstruct the plant RNA silencing defense pathway by seizing small RNAs to inhibit uploading into RNA-induced silencing complexes. NSs also interacts with several host RNA silencing pathway components [5]. Similar to findings for TSWV and INSV, we did not detect any homotypic or heterotypic viral protein interactions involving the IYSV NSs protein by BiFC. Recently, it was reported that the predicted hairpin structure of TSWV RNA, found between the N and NSs ORFs on the S segment, plays a role in translation in concert with viral N protein [40]. We could not detect any interaction between IYSV N and NSs proteins, the reason for which might be the requirement of the RNA hairpin structure for association or because of differences between the two viruses.

Tospovirus glycoproteins have been shown to be critical for transmissibility by thrips vectors [15]. Reportedly, TSWV G_N_ and G_C_ cause membrane deformation and interact with each other and with the N protein to help in particle assembly at the Golgi complex [9, 41]. The maturation mechanism of glycoproteins and its interplay with endomembrane systems has recently been reported [42]. TSWV G_C_ solely localizes in ER when transiently expressed in *N*. *tabacum* protoplasts, whereas G_N_ was found both within the ER and Golgi membranes [42]. During maturation of TSWV, G_C_ was shown to localize at ER membranes. However, it becomes ER export competent upon co-expression with G_N_ [42]. Nucleocapsid protein recruits ER-resident protein and interacts with cytosolic tail of G_C_ at the ER export sites (ERESs) for its transport to the Golgi [42–43]. IYSV G_N_ and G_C_ localized in the ER network. IYSV G_C_ also localized to the nucleus. Similar pattern of localization of G_N_ and G_C_ was observed in INSV [23]. We did not observe any interaction between IYSV N and G_N_ or G_C_ by BiFC in infected plants. Low-level expression of glycoproteins or steric constraints preventing the YFP halves from coming together may have been responsible for the observed lack of BiFC interaction between N and glycoproteins [23]. Further investigation on the behavior of glycoproteins will provide a better insight into their interaction with the N protein. Additionally, based on previous reports and this study, it could also be concluded that tospovirus N protein forms a motile multimeric complex that moves from cytoplasm to cell periphery through actin/ER network, where it possibly interacts with the NSm protein and glycoproteins to facilitate the movement and assembly of virus particles during infection process. The movement of the N protein is initiated in the presence of replicating virus and may involve some host factors. In summary, our studies showed the localization and interactions of transiently expressed IYSV proteins in *N*. *benthamiana* plants. A minor shift in the localization of IYSV N protein in infected plants was noticed compared to uninfected plants. Interaction studies of IYSV proteins were conducted by BiFC in uninfected and infected plants. The positive BiFC interactions of IYSV N and NSm proteins were validated by pull down and yeast-2-hybrid assays to reduce the chances of any false positives. In addition, the critical regions responsible for IYSV N/N, N/NSm, and NSm/NSm interactions were identified. More detailed investigation of the molecular basis of these interactions would facilitate a better understanding of the replication, transcription, and assembly processes in tospoviruses.

## Supporting Information

S1 Fig High-resolution confocal micrographs showing the localization of *Iris yellow spot virus* (IYSV) proteins in epidermal cells of IYSV-infected transgenic *Nicotiana benthamiana* plants containing red endoplasmic reticulum marker (ER-RFP). Confocal micrographs represent IYSV fusion proteins to the C-terminus of green fluorescent protein (GFP). Columns from left to right show GFP-gene fusion (1), ER-RFP (2), and the overlay of the images (3). (a-c) GFP-IYSV N co-expression with ER-RFP; (d-f) GFP-IYSV NSm co-expression with ER-RFP; (g-i) GFP-IYSV NSs co-expression with RFP-H2B and ER-RFP; (j-l) GFP-IYSV G_N_ co-expression with ER-RFP; (m-o) GFP-IYSV G_C_ co-expression with ER-RFP. Each micrograph represents a minimum of 50 cells that were examined for localization. Scale bar = 20μm.
(TIF)

S2 Fig Confocal micrographs to show the interaction of *Iris yellow spot virus* (IYSV) nucleocapsid (N) and movement (NSm) proteins in epidermal leaf cells of transgenic *Nicotiana benthamiana* (CFP-H2B) plants by bimolecular fluorescence complementation (BiFC) assay. Shown are the images of fluorescence emitted by YFP+CFP (left), transmitted light mode (middle) and a merge of all panels (overlay; right). The constructed clones were agroinfiltrated in pairwise combination in infected CFP-H2B plants as follows: (a) wild-type uninfected; none, (b) none, (c) ER-CFP marker only, (d) nYFP-N1+cYFP-N1, (e) N-nYFP+N-cYFP, (f) N-nYFP+cYFP-N1, (g) nYFP-N1+N-cYFP-N1, (h) NSm-nYFP+NSm-cYFP, (i) NSm-nYFP+cYFP-N1, (j) nYFP-N1+NSm-cYFP, (k) N-nYFP+NSm-cYFP, (l) NSm-nYFP+N-cYFP, (m) nYFP-C1+cYFP-C1, (n) nYFP-N+cYFP-N, (o) nYFP-N+cYFP-C1, (p) nYFP-C1+cYFP-N, (q) nYFP-NSm+cYFP-NSm, (r) nYFP-NSm+cYFP, (s) nYFP+cYFP-NSm, (t) nYFP-NSm+cYFP-N, (u) nYFP-N+cYFP-NSm, (v) nYFP-NSm+N-cYFP. (w) nYFP-N+NSm-cYFP. Co-expression of (n), (q), (t) and (u) showed positive BiFC signal (YFP fluorescence). *Interaction was tested with the same proteins (N/N, Nsm/Nsm). Each micrograph represents a minimum of 50 cells that were examined. Scale bar = 20μm.
(TIF)

S3 Fig *In planta* interactions of *Tomato spotted wilt virus* proteins as examined by bimolecular fluorescence complementation (BiFC) assay. Interaction assays were performed in leaf epidermal cells of transgenic *Nicotiana benthamiana* plants expressing cyan fluorescent protein fused to the nuclear marker histone 2B (CFP-H2B), and cyan endoplasmic reticulum (ER-CFP) marker. Column 1 shows BiFC, column 2 shows localization of CFP-H2B and ER-CFP (nucleus and ER), and column 3 shows a merge of all panels (overlay). The first and second proteins mentioned in each pair of interactors were expressed as C-terminal fusions to the amino-terminal half of YFP and as C-terminal fusions to the carboxy-terminal half of YFP respectively. A set of positive interactions is shown here after testing interactions in all pairwise combinations: (a-c) N/N, (d-f) NSm/ NSm, (g-i) NSm/N. Each micrograph represents a minimum of 50 cells that were examined. Scale bar = 20μm.
(TIF)
