# Supplementary figures and images for: In Vivo Localization of Iris yellow spot Tospovirus (Bunyaviridae)-Encoded Proteins and Identification of Interacting Regions of Nucleocapsid and Movement Proteins
Source: PLoS One. 2015 Mar 17;10(3):e0118973. doi: 10.1371/journal.pone.0118973 (PMC4363525; doi:10.1371/journal.pone.0118973)

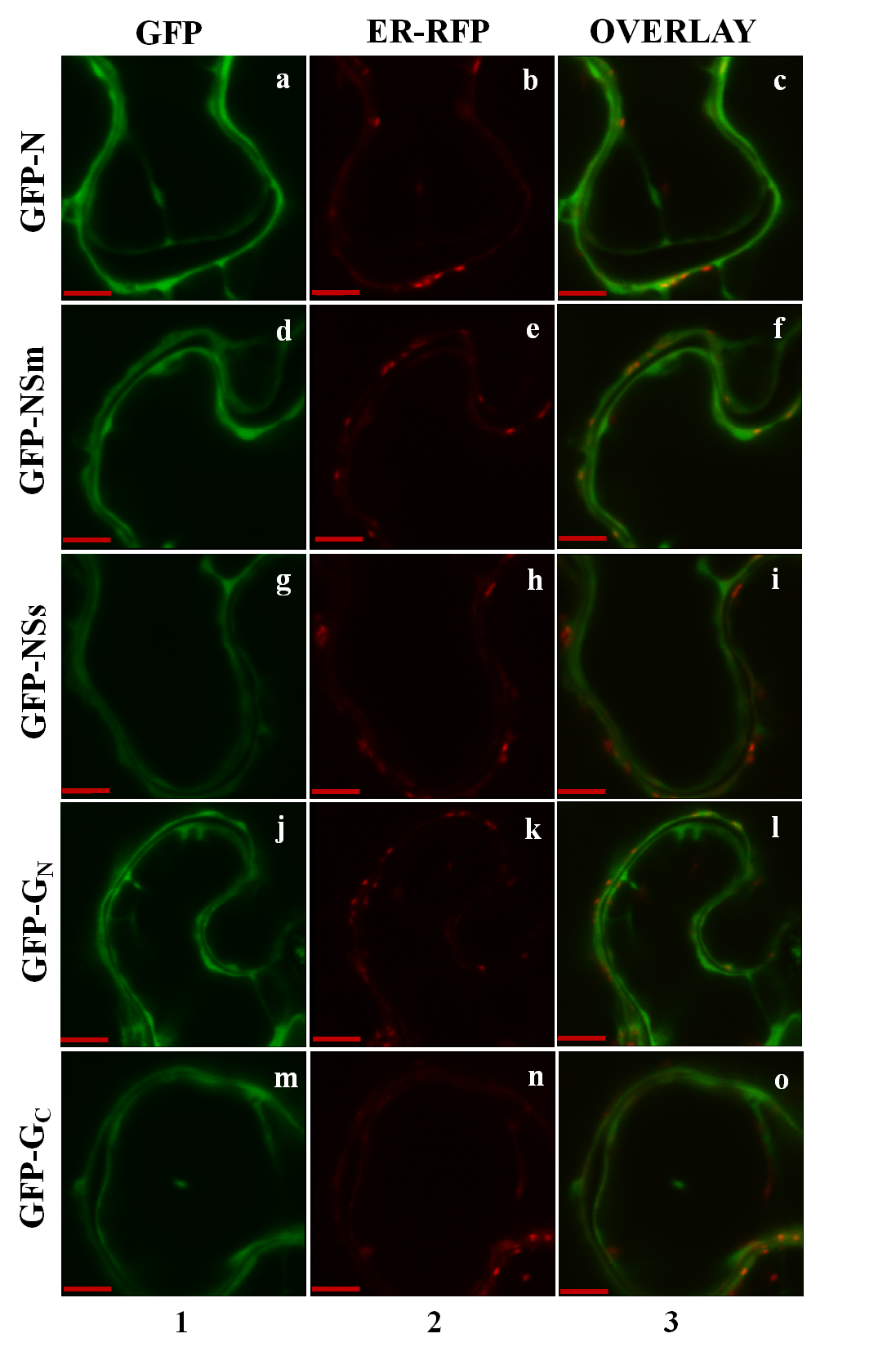

Supplement: S1 Fig — Confocal micrographs represent IYSV fusion proteins to the C-terminus of green fluorescent protein (GFP). Columns from left to right show GFP-gene fusion (1), ER-RFP (2), and the overlay of the images (3). (a-c) GFP-IYSV N co-expression with ER-RFP; (d-f) GFP-IYSV NSm co-expression with ER-RFP; (g-i) GFP-IYSV NSs co-expression with RFP-H2B and ER-RFP; (j-l) GFP-IYSV GN co-expression with ER-RFP; (m-o) GFP-IYSV GC co-expression with ER-RFP. Each micrograph represents a minimum of 50 cells that were examined for localization. Scale bar = 20μm. (TIF) [file pone.0118973.s001.tif]

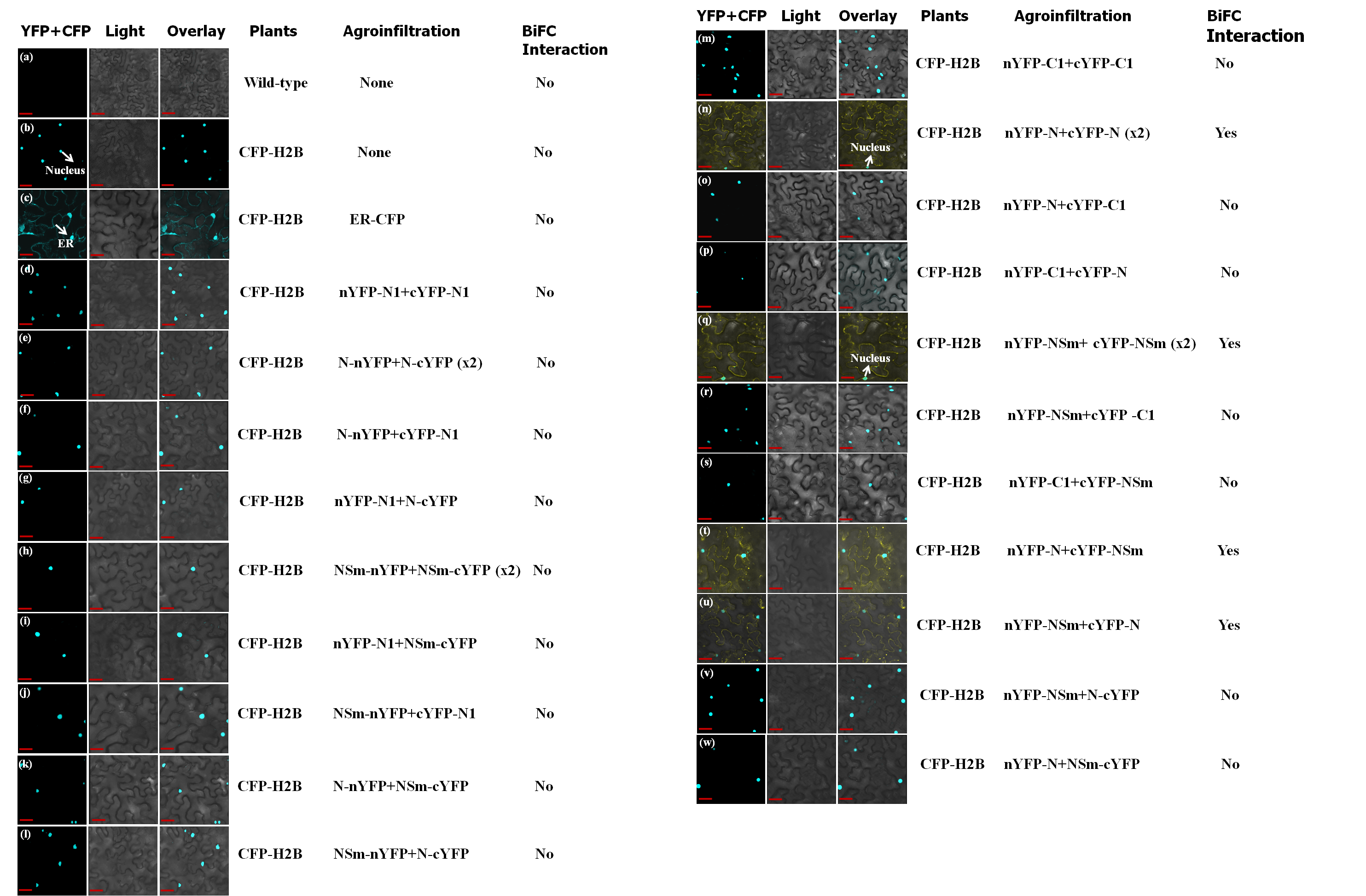

Supplement: S2 Fig — Shown are the images of fluorescence emitted by YFP+CFP (left), transmitted light mode (middle) and a merge of all panels (overlay; right). The constructed clones were agroinfiltrated in pairwise combination in infected CFP-H2B plants as follows: (a) wild-type uninfected; none, (b) none, (c) ER-CFP marker only, (d) nYFP-N1+cYFP-N1, (e) N-nYFP+N-cYFP, (f) N-nYFP+cYFP-N1, (g) nYFP-N1+N-cYFP-N1, (h) NSm-nYFP+NSm-cYFP, (i) NSm-nYFP+cYFP-N1, (j) nYFP-N1+NSm-cYFP, (k) N-nYFP+NSm-cYFP, (l) NSm-nYFP+N-cYFP, (m) nYFP-C1+cYFP-C1, (n) nYFP-N+cYFP-N, (o) nYFP-N+cYFP-C1, (p) nYFP-C1+cYFP-N, (q) nYFP-NSm+cYFP-NSm, (r) nYFP-NSm+cYFP, (s) nYFP+cYFP-NSm, (t) nYFP-NSm+cYFP-N, (u) nYFP-N+cYFP-NSm, (v) nYFP-NSm+N-cYFP. (w) nYFP-N+NSm-cYFP. Co-expression of (n), (q), (t) and (u) showed positive BiFC signal (YFP fluorescence). *Interaction was tested with the same proteins (N/N, Nsm/Nsm). Each micrograph represents a minimum of 50 cells that were examined. Scale bar = 20μm. (TIF) [file pone.0118973.s002.tif]

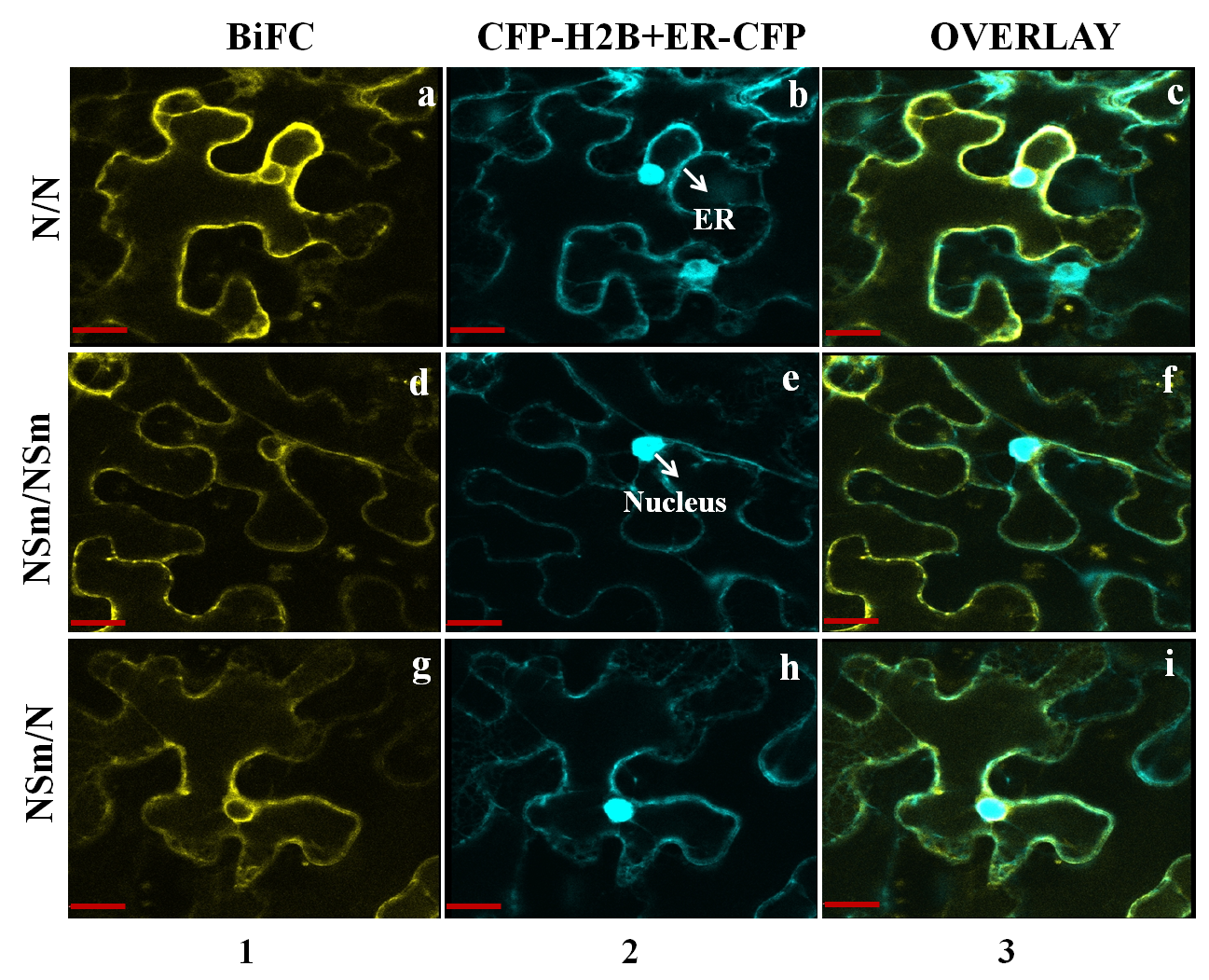

Supplement: S3 Fig — Interaction assays were performed in leaf epidermal cells of transgenic Nicotiana benthamiana plants expressing cyan fluorescent protein fused to the nuclear marker histone 2B (CFP-H2B), and cyan endoplasmic reticulum (ER-CFP) marker. Column 1 shows BiFC, column 2 shows localization of CFP-H2B and ER-CFP (nucleus and ER), and column 3 shows a merge of all panels (overlay). The first and second proteins mentioned in each pair of interactors were expressed as C-terminal fusions to the amino-terminal half of YFP and as C-terminal fusions to the carboxy-terminal half of YFP respectively. A set of positive interactions is shown here after testing interactions in all pairwise combinations: (a-c) N/N, (d-f) NSm/ NSm, (g-i) NSm/N. Each micrograph represents a minimum of 50 cells that were examined. Scale bar = 20μm. (TIF) [file pone.0118973.s003.tif]
